# Molecularly Resonant Metamaterials for Broad‐Band Electromagnetic Stealth

**DOI:** 10.1002/advs.202301170

**Published:** 2023-04-21

**Authors:** Yifan Wang, Jiarong Niu, Xin Jin, Xiaoming Qian, Changfa Xiao, Wenyu Wang

**Affiliations:** ^1^ State Key Laboratory of Separation Membranes and Membrane Processes School of Materials Science and Engineering Tiangong University Tianjin 300387 China; ^2^ State Key Laboratory of Separation Membranes and Membrane Processes School of Textile Science and Engineering Tiangong University Tianjin 300387 China; ^3^ Fiber Materials Research Center Shanghai University of Engineering Science Shanghai 201620 China

**Keywords:** metamaterials, molecular structure of polypyrrole, split resonant rings, wide‐band electromagnetic stealth

## Abstract

Electromagnetic (EM) metamaterial is a composite material with EM stealth properties, which is constructed by artificially reverse engineering metal split resonance rings (SRR). However, the greatest limitation of EM metamaterials is that they can only stealth at a fixed and lower frequency of EM waves, and modern processing techniques still cannot meet the accuracy requirements to fabric nano‐size structural unit. Nano‐sized and even ultra‐small SRR at molecular level are promising arrays to realize the ability of EM stealth function at a higher frequency, although it has proven challenging to synthesize long, straight, connected molecular SRR, and also difficult to arrange those molecular SRR into a strict array. Here, the study overcomes this challenge and demonstrates that the fabric of polypyrrole molecular SRR achieves an ultra‐small inner diameter of 2.49 Å and realizes the arrays arrangement at molecular level. Furthermore, the study exploits the EM stealth function and verifies that such arrays of molecular SRR with 2.49 Å have the ability to reach high‐performance EM stealth in the range of 10^6^–10^16^ Hz. This design concept opens a pathway for developing new metamaterials with broadband EM wave stealth and also serves the wider range of new applications.

## Graphical abstract

A challenging molecular‐level design idea for broad‐band electromagnetic (EM) stealth materials put forward. This breaks the limitation that modern technology cannot machine rings with the size of nanometres in traditional inverse engineering. The fabrics based on the array of polypyrrole molecular rings with 2.49 Å diameter can achieve EM stealth in the range of 10^6^‐10^16^ Hz.

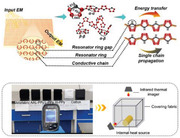

## 1 Introduction

As the most magical color and the most exotic electromagnetic (EM) properties in the 21st century, metamaterials have become an exciting frontier science field of physics, material science, engineering, and chemistry.^[^ ^1^, ^2^ ^]^ However, up to now, all metamaterials (e.g.,EM metamaterials,^[^ ^3^, ^4^ ^]^ optical metamaterials,^[^ ^5^, ^6^ ^]^ mechanical metamaterials and other metamaterials with unnatural properties) still rely on the collection of artificially designed microstructure units.^[^ ^7^, ^8^, ^9^, ^10^ ^]^ Taking EM metamaterials as an example,^[^ ^11^, ^12^ ^]^ the EM metamaterials can produce fascinating physical properties unavailable in naturally when permittivity and permeability of the material are simultaneously negative.^[^ ^13^, ^14^, ^15^, ^16^, ^17^, ^18^ ^]^ These fascinating properties can allow metamaterials to be widely used in various fields such as wireless communication,^[^ ^19^, ^20^ ^]^ radar stealth,^[^ ^21^, ^22^ ^]^ and thermal energy conversion.^[^ ^23^, ^24^ ^]^ Metamaterial technology can customize macroscopic physical field responses only through fine design of structural dimensions, which subverts the traditional natural material system's need to study component properties and find appropriate material synthesis methods, achieving on‐demand reverse design based on macroscopic physical field cognition. This magical feature is derived from the interaction of between artificial metal split resonant rings (SRR) arrays and EM wave, in which SRR are calculated according to the wavelength of working EM waves based on inverse engineering.^[^ ^25^, ^26^ ^]^ That is to say, it is necessary to calculate the unit size, spacing distance and opening size of SRR according to the fixed working EM wave length,^[^ ^27^, ^28^ ^]^ resulting in the most EM metamaterials can only resonate and produce stealth function under EM wave in a narrow range or fixed frequency.^[^ ^29^, ^30^, ^31^, ^32^ ^]^

In response to this limitation, some novel EM metamaterials have been developed to achieve broadband EM work via combining several resonators of different sizes or vertically stacking multi‐layer subwavelength metal structures.^[^ ^33^, ^34^, ^35^ ^]^ Such as metamaterial of concave ring complementary structure resonator proposed by Huang et al.,^[^ ^36^ ^]^ metamaterial of four strip resonators with different resonant modes proposed by Zhao et al.,^[^ ^37^ ^]^ and metamaterial of single circular sector resonator proposed by Luo et al..^[^ ^38^ ^]^ Despite improving the EM stealth of metamaterials over a range of frequencies, only for EM waves at low frequencies, resulting in a dire need for structure of SRR to match wider and higher EM wave frequencies.

Generally, the size of the SRR cell should be designed to be much smaller than the operating wavelength. This makes it difficult to fabricate resonant rings with nano or even smaller dimensions using current processing techniques if metamaterials are required to exhibit stealth effects at higher frequencies. Therefore, it is a big challenge for a scientist to design advanced EM metamaterials with stealth function at higher and broader wave frequency.^[^ ^27^, ^39^, ^40^, ^41^, ^42^ ^]^

To address this challenge, various size of resonant rings were designed and fabricated according to inversed calculating. The most classical metamaterials for SRR reaches 3.3 mm to accommodate EM waves below 8.5 × 10^9^ Hz (with wavelengths of 35 mm) in 2006.^[^ ^29^ ^]^ In 2023, the metamaterial unit structure composed of double‐groove rectangular annular patches, with a size of 3.05 mm × 2.85 mm, can achieve resonance at the frequency of 28 × 10^9^ Hz.^[^ ^43^ ^]^ To date, the minimum diameter size of SRR unit can reach 500 nm by nanoimprint technology,^[^ ^44^ ^]^ which can only adapt to far‐infrared EM wave with wavelengths above 10 µm (about the frequency of 10^12^ Hz). To qualify the stealth function at EM wave with higher frequency or shorter wavelengths, the sizes of SRR units should not only be ultra‐small (nanometer or even smaller molecular size), but also perfectly aligned,^[^ ^19^, ^45^ ^]^ which is almost impossible to achieve current processing technology.^[^ ^23^, ^46^, ^47^, ^48^, ^49^, ^50^, ^51^ ^]^

Here, we proposed a novel EM metamaterial with molecular‐level SRR of 2.49 Å diameter for broadband EM wave stealth using the unique molecular structure of polypyrrole (PPy). PPy is a conductive polymer that is widely used in the fields of sensing,^[^ ^52^, ^53^, ^54^ ^]^ electrocatalysis,^[^ ^55^ ^]^ and manufacturing electrodes due to its excellent electrical conductivity.^[^ ^56^, ^57^, ^58^ ^]^ It has three linkages structures, namely, *α*‐*α*, *α*‐*β*, and *β*‐*β* linkages. In *α*‐*α* linkage, it can be found that C—N—C forms a straight chain unit. In order for this straight chain to be in a plane, C=C—C=C conjugation ensures that C—N—C is in a plane, so that the circular unit composed of C=C—C=C enables each repeating unit to be in a plane, while keeping C—NH—C in a plane while maintaining the straight chain. This can only be satisfied simultaneously in the *α*‐*α* linkage of PPy.

By polymerization reactions under low temperature ^[^ ^59^ ^]^ and induction of hydrogen bonding, we addressed the issue of both the continuity of *α*‐*α* conjugate bond of the molecular chain and the co‐planarity of each molecular chain, leading to an orderly arrangement of all linear molecular chains in the same plane. In order to further improve the resonance effect of molecular SRR arrays with EM wave frequencies, multiwall carbon nanotubes (MWCNTs) were added as energy transfer bridges during the polymerization. These linearly arranged molecular chains can adaptively adjust their vibration based on the vibration of the external operating frequency, which results in the resonance rings on all molecular chains always maintaining the same frequency vibration as the external operating frequency, thereby achieving a wide band operating frequency. These super small molecular resonant rings were structurally designed like arrays in a connected net, which guarantee the resonant rings can work together efficiently at same frequency according to the working wave, and therefore demonstrated excellent stealth function of EM wave in the range of 10^6^–10^16^ Hz. The design of this resonant ring is based on a molecular‐level design that has been unprecedented in other previous studies.

## 2 Results and Discussion

### 2.1 Design of Arrays with Molecular SRR

**Figure 1:**
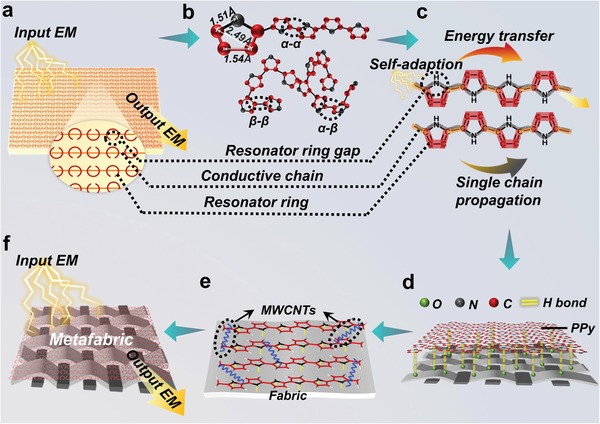
Schematic diagram of arrays of molecular SRR. a) Resonant ring and conductive metal wire model of traditional EM metamaterials. b) Three linkage modes of PPy. c) Planar molecular resonant rings in one PPy chain polymerized at low temperature. d) Coplanar of molecular resonant rings in multi‐chains of PPy polymerized at low temperature combined hydrogen bond strategy. e) Array of connected coplanar molecular resonant rings polymerized at low temperature combined hydrogen bond strategy with MWCNTs as resonant bridge. f) Schematic diagram of metafabric with stealth function.

To implement the metamaterials specification in the above molecular resonant rings, the molecular structure with conductive chain, conductive split unit, and appropriate spatial structure of the polymer are well‐designed according to our hypothetic idea shown in **Figure** **1**. At the molecular structure‐level, the *π*‐conjugated conductive polymer PPy was scientifically analyzed, in which the five‐membered ring structure of PPy had the surprisingly similar structure of the metal SRR (Figure 1a,b). Importantly, the size of this conductive ring is at the molecular level (inner diameter: 2.49 Å, the opening distance: 2.44 Å; Figure 1b),^[^ ^34^ ^]^ it is almost equivalent to the wavelength of X‐rays (*λ* = 0.1–10 Å). Therefore, it is theoretically possible to design EM metamaterials composed of molecular resonant ring unit capable of stealth at high‐frequency EM wave (which can reach X‐rays frequency range).

However, compared with the arrays of metallic resonant rings, controlling the spatial arrangement of molecular resonant rings during polymerization is much more difficult. This difficulty mainly comes from pyrrole has three polymerization sites, including *α*‐*α*, *α*‐*β* and *β*‐*β* linkage modes, which results in an irregular random disorder arrangement of the resonance rings of the PPy molecule, so it exhibits no‐planar property (Figure 1b). To obtain molecular resonant ring array positioned in a planar connected net, three difficulties need to be overcome: 1) At the single‐chain level, one straight and long PPy *α*‐*α* chain is necessary. 2) At the multi‐chains level, molecular resonance rings from different chains should be formed arrays in one plane. 3) At the inter‐chains level, the chains should be effectively connected to each other to ensure consistency with the operating frequency.

Only by solving the above three difficulties, the metamaterial designed according to molecular resonant ring principle can quickly respond to the operating frequency, and produce a resonance effect to achieve stealth function. We approached the preparation of planarized and regularized molecules resonance ring networks on the cotton fabric surface by using hydrogen bonding‐guided and low‐temperature polymerization methods. To make a clear understanding the relationship between the structure of PPy and the performance, the polymerization was processed step by step.

First, pyrrole polymerized on fabric at low temperature (L‐PPy fabric). Py polymerizes at low temperature to produce more *α*‐*α* linkages.^[^ ^60^ ^]^ The *α*‐*α* linkage promotes the formation of planarized *π*‐conjugate bonds, which makes the molecular chains less tangled and bent and more facilitates the propagation of EM wave along the main chain (Figure 1c). Although we have constructed planar arrays of molecular resonant ring on single molecule chain, the intertwining of different molecular chains results in molecular resonant rings on various polymer chains that are still disordered.

Second, polyacrylic acid (PAA) is introduced into the fabric for pyrrole polymerization (AAL‐PPy fabric), which realizes the combination of hydrogen bonding strategy and low‐temperature effect. Importantly, taking advantage of the orientational nature of hydrogen bonding, PPy molecular resonance rings from various PPy chains are likely to arrange as high order array in one plane on fabrics (Figure 1d), which would help to resonant together to the working wave. At this point, all linear molecular chains are driven by five‐membered rings (resonator rings) arranged in an orientation, which can adaptively adjust its vibration frequency according to the external working frequency.

Thirdly, to further improve the molecular rings on all different molecular chains vibrate at the same frequency, MWCNTs were brought as energy transfer bridges in the polymerization (Figure 1e). Therefore, the PPy fabric manufactured by this advanced polymerization method (Metafabric) has the interconnected and structurally planar array with super‐small molecular resonant rings, which is ready to stealth at broad‐band wave based on the design of molecular resonant rings (Figure 1f).

### 2.2 Characterization of PPy Molecular SRR

**Figure 2:**
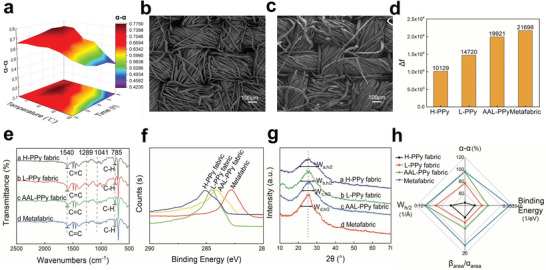
Characterization of PPy arrays of molecular SRR. a) Effect of time and temperature on *α*‐*α* linkage the relationship of PPy. b) SEM image of the cotton fabric (scale bar: 100 µm). c) SEM image of the metafabric (scale bar: 100 µm). d) FTIR results of different polymeric fabrics and their extreme Δ*f* values. e) FTIR results of different polymeric fabrics. f) The C1s XPS spectra of polymeric fabrics under different conditions. g) XRD patterns of polymeric fabrics under different conditions. h) By *α*‐*α* content, binding energy, *β*_area_/*α*_area_, and half peak width (W_h/2_) radar images of different polymeric fabrics.

More *α*‐*α* linkages of PPy are beneficial for the formation of planarized *π*‐conjugate bonds and therefore formed orderly arrangement of molecular SRR. The content of *α*‐*α* linkages of PPy is strongly dependent on the condition of polymerization. First, the effect of reaction temperature and time on the content of *α*‐*α* linkages was investigated by the FTIR absorption peak intensity ratio at 785 cm^−1^ and 1540 cm^−1^ (*I*_785_/*I*_1540_) (Figure S1 and Table S1, Supporting Information) and the percentage of *α*‐*α* linkages were summarized in **Figure** **2**a.^[^ ^61^, ^62^, ^63^ ^]^ The *α*‐*α* linkage content of PPy polymerized at high temperature (H‐PPy) is 48.25%, while data of PPy polymerized at low temperature (L‐PPy) increased to 77.43%, indicating the lower temperature and longer reaction time encourage to form higher *α*‐*α* linkage content of PPy.

Figure 2b,c shows that PPy coating cotton fabric were uniform without agglomeration.

Furthermore, the hydrogen bond involving in pyrrole polymerization is an effective way to align PPy chains into ordered connection. The results of XPS in Figure S2 (Supporting Information) showed that the content of ‐COOH groups on fabric after PAA treated was increased obviously, which are the active sites and ready to establish hydrogen bond with ‐NH of PPy. The force constant ∆f calculated from data of FTIR in Figure S3 (Supporting Information) associated with molecular interaction between ‐COOH and ‐NH are shown in Figure 2d, indicating that strong hydrogen bond interaction was established for metafabric.

In addition, the established hydrogen bonds have the function of guiding PPy to form *α*‐*α* linkage. The content of *α*‐*α* linkage of four samples, H‐PPy, L‐PPy, AAL‐PPy, and metafabric, is studied by FTIR, XPS, and XRD, respectively (Figure 2e–h). In the FTIR of different polymer fabrics shown in Figure 2e and Table S2 (Supporting Information), the ratio of *I*_785_/*I*_1540_ of AAL‐PPy fabric and metafabric increased significantly to 95.79% and 96.01%, which is associated with the forming hydrogen bond. Concomitantly, hydrogen bond promotes the forming of *α*‐*α* linkage PPy can be further confirmed from the data XPS spectra by analysis of the shift of C1s and the peak area ratio of *α*‐CH and *β*‐CH (*β*_area_/*α*_area_) (Figure 2f). The more regular the molecular chains are, the lower the binding energy of C. It can be clearly seen from the Figure 2f that the displacement of C1s peak and the C binding energy of metafabric shift to the lowest binding energy, indicating that its molecular chain arrangement is more regular. Meanwhile, the peak area ratios of *β*_area_/*α*_area_ was calculated using the split‐peak fitting technique, and the larger *β*_area_/*α*_area_ of metafabric showed the more content of the *α*‐*α* linkage in the PPy (Figure S4 and Table S3, Supporting Information).^[^ ^64^ ^]^

The peak width of the XRD spectra clearly demonstrates that hydrogen bonding polymerization promotes the degree of order of PPy (Figure 2g). The peak of PPy at 25° is wide for the sample of H‐PPy and L‐PPy, while the peak became sharper for AAL‐PPy and metafabric. Therefore, experimental data from FTIR, XPS, and XRD are summarized in Figure 2h, pointing that lower temperature combined hydrogen bond polymerization strategy contributes the higher *α*‐*α* linkage with ordered PPy molecular chainsstructure.

### 2.3 Electromagnetic Properties in Lower Frequency

**Figure 3:**
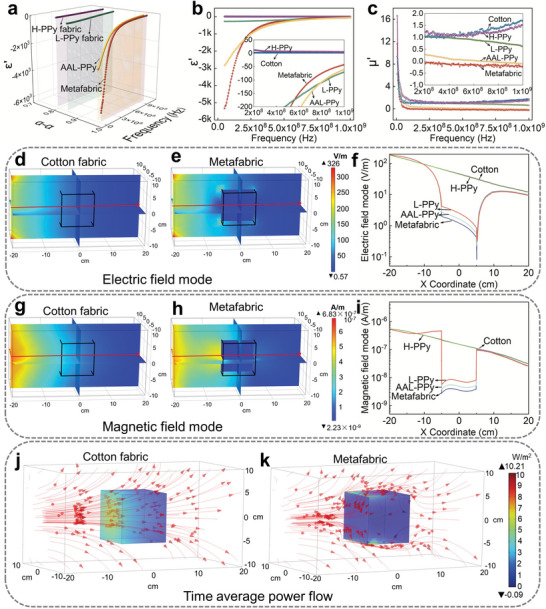
EM property and stealth simulation of metafabric. a) The relationship between *α*‐*α* content and permittivity of four kinds of PPy fabrics at different frequencies. b) The permittivity of four kinds of PPy fabrics and cotton fabric change with frequency. c) The permeability of four kinds of PPy fabrics and cotton fabric varies with frequency. d,e) The change of 3D electric field mode of cotton fabric and metafabric in COMSOL. f) The change of electric field mode strength of four PPy fabrics and cotton fabric along the path shown in (d) and (e) at 1 × 10^9^ Hz. g,h) The change of 3D magnetic field mode of cotton fabric and metafabric in COMSOL. i) The change of magnetic field mode strength of four PPy fabrics and cotton fabric at 1 × 10^9^ Hz. j,k) Time average power flow of cotton fabric and metafabric.

To understanding the effect of molecular resonant structure on the EM properties of the obtained sample fabrics, the frequency dispersion of permittivity and permeability were measured shown in (**Figure** **3**a–c). The change of *ε* with content of *α*‐*α* linkage and frequency of working wave is shown in Figure 3a,b, respectively. The results exhibit that permittivity behavior strongly depends on the content of *α*‐*α* linkage of PPy, in which the permittivity of H‐PPy (content of *α*‐*α* linkage is 44.65%) are positive in the whole range of testing frequency, while the permittivity of L‐PPy, AAL‐PPy, and metafabric, possessing higher percentage of *α*‐*α* linkage as 76.64%, 95.79%, and 96.01%, respectively, showed obvious negative property. Therefore, the negative permittivity behavior stem from the plasma oscillation of delocalized electrons in the conductive polymer chains with higher content of *α*‐*α* linkage.^[^ ^65^, ^66^ ^]^

Figure 3c illustrates the permeability of sample fabrics with different percentage of *α*‐*α* linkage. The data of measurement showed that permeability has the relationship with the content of *α*‐*α* linkage. Compared with the H‐PPy fabric, the permeability of L‐PPy fabric and AAL‐PPy fabric decreased with an increase in the content of *α*‐*α* linkage. Notably, although the higher percentage of *α*‐*α* linkage is beneficial to the decrease of permeability values of sample fabric, the permeability values of L‐PPy fabric and AAL‐PPy fabric are still positive. Interestingly, the permeability values of metafabric dropped to the negative value obviously even the content of *α*‐*α* linkage of metafabric is maintain the same level with the AAL‐PPy fabric. Based on the real testing result, the negative permeability was attributed to the molecular rings from all different molecular chains of PPy can vibrate at the same frequency associated with the connected and structurally planar array of molecular resonant rings.^[^ ^67^ ^]^ This just shows that only metafabric has a more complete resonant ring structure. Therefore, the experiment verified that metafabric with both the permittivity and permeability are simultaneously negative and are successfully fabricated based on the design idea of molecular resonant rings. The metafabric is a flexible and molecular size‐based resonant ring design. It can adaptively adjust its resonant frequency according to the external working frequency to meet the application in a wide band range. This is beyond the reach of previous work. (**Table** **1**)

**Table 1:** Compare the research results of this work with similar research results

Material	Resonant ring size [mm]	Working frequency [GHz]	Special performance	Reference
UPEF‐BN/PU	4 × 10^−5^	10^2^‐10^3^ (300‐1100)	/	[70]
Cu/FR‐4	3.5	10^0^ (3.4‐3.8 7.0‐7.5)	Negative‐index	[71]
GO	1.1 × 10^−2^	10^3^‐10^4^ (9890‐16770)	High incidence angle	[72]
Cu/FR‐4	7.2	10^0^ ‐10^1^ (8‐27)	Insensitive to polarization	[73]
Cu/PI	26	10^0^ ‐10^1^ (2‐12)	Polarization insensitive Good absorption for a wide incidence angle	[74]
Cu/FR‐4	10	10^0^ ‐10^1^ (4‐12)	Perfect absorption >40dB shielding effect in the tested frequency band	[75]
Cu/FR‐4	5.6	10^0^ ‐10^1^ (8‐27)	Wide angle stability Polarization insensitive	[76]
Cu/FR‐4	9	10^0^ (3.52‐3.79)	Negative refractive index	[77]
Cu/PI	8.6 × 10^−2^	10^2^‐10^3^ (250‐1000)	Active Tunable Multifrequency	[78]
Cu	11	10^0^ ‐10^1^ (2‐12)	Negative permittivity	[79]
MF/Mxene/PEG	PCM	10^0^ (1.8‐2.6)	High latent heat storage	[80]
GO@PPy/CIF	Irregular powder	10^0^ ‐10^1^ (2‐18)	/	[81]
Ti_3_C_2_TXMXene	Irregular powder	10^1^ (13.4‐17.7)	/	[82]
Finite element simulation	2.85	10^1^ (27.48‐30)	Negative permittivity and Permeability	[43]
Cu/FR‐4	8	10^0^ (2.46‐4.99 8.06‐10.02)	Negative permittivity and Permeability	[83]
Cu/FR‐4	9.7	10^0^ (6.2‐7.0) 10^0^ ‐10^1^ (8.7‐9.0 11.3‐11.6)	Negative permittivity and Permeability	[84]
Cu/PI	0.16	10^2^ (242‐254)	Negative permittivity and Permeability	[85]
Water based metamaterials	14	10^1^ (12.5‐22.7)	Negative permittivity and Permeability	[86]
PPy/MWCNTs	Irregular powder	10^−1^‐10^0^ (0.865‐1)	Negative permittivity and Permeability	[67]
PPy/MWCNTs	2.49 × 10^−7^	10^−3^‐10^7^	Negative permittivity and Permeability Ultrawide band electromagnetic stealth	This work

In order to have a deeper understanding of the propagation trajectory of EM wave through fabrics with different EM properties, we chose COMSOL Multiphysics finite element for the simulation (Figure S5‐S8, Supporting Informatioin). Firstly, the distribution of intensity variation across fabric cube were stimulated in the electric and magnetic fields, respectively. As shown in the mapping images of electric and magnetic fields (Figure 3d,e,g,h), the most obvious difference is that the cotton fabric exhibits the continuous attenuation of intensity in both electric and magnetic field (from left to right, orange yellow gradually changes to dark blue), while the metafabric, which is obviously in magnetic field, shows cliff attenuation to dark blue. In order to show the change of field strength more clearly, the simulated values of electric field and magnetic field on the central axis of the whole field are extracted. The central axis passes through the body center of the fabric cube, which is indicated by the red line in Figure 3d‐e,g‐h. The simulation values of cotton fabric in both electric and magnetic field decreased smoothly. Compared with the cotton fabric, with the spatial arrangement of molecular resonance rings trends to be perfected, especially in the case of metafabric has a sudden plateau dropping in strength value, followed by rapid return to the strength values of cotton fabric (Figure 3f,i and Figures S6 and S8, Supporting Information). This is due to the dual negative EM properties of the metafabric, which has a special EM stealth function. Meanwhile, we still studied the spatial distribution of energy changing in time through the propagation trace of the time average power flow (Figure 3j,k). The simulation result can be fully demonstrated that how smoothly the waves wrap around metafabric cube and propagate to the far side, as if the waves had never passed through anything at all (Figure S9, Supporting Information). This phenomenon is highly consistent with the trend in the result for EM materials parameters.

Due to the limitation of the existing testing methods, the electric permittivity and permeability can be really tested in the range of 1 MHz–1 GHz. In order to demonstrate the EM stealth function of the metafabric in a broader wavelength rang, we investigated the behavior of metafabric under radiated EM wave in the form of thermal shielding. It is well known that heat can be delivered by convection, radiation, and thermal conduction. Unlike the mechanism of conduction and convection, radiation propagates in the form of EM wave. Three heat transfer experiments have been designed to understand the heat transfer capacity of different fabrics under the three heat transfer methods. The results show that there is no significantly different between metafabric and other fabrics in terms of heat conduction and convection capacity (Figure S10 and Table S4, Supporting Information).

### 2.4 EM Stealth Performance in Higher Frequency

**Figure 4:**
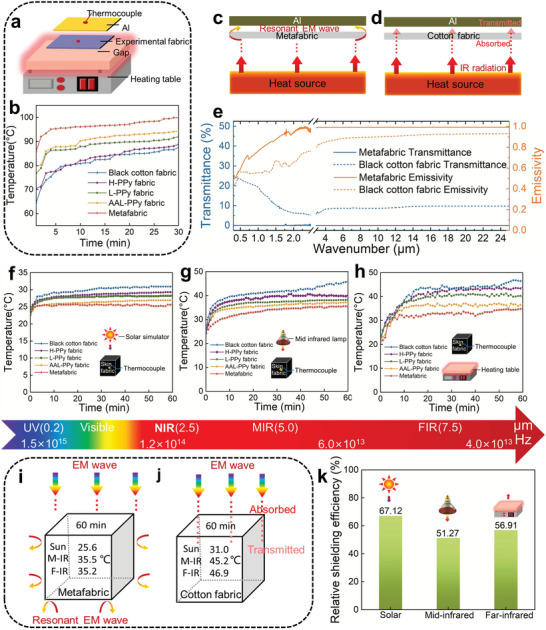
The stealth performance of the metafabric under EM wave with different frequency. a) Model diagram of baffle heating experiment. b) Time temperature curve of baffle heating experiment for four kinds of PPy fabrics and cotton fabric. c,d) In the baffle experiment, schematic diagram of thermal radiation wave propagation on fabrics surface. e) Reflectivity and emissivity spectra of the metafabric at wavelengths ranging from 0.2 to 25 µm. f–h) Curve of the internal temperature of the fabrics cubes as a function of time when the fabric is heated externally by different heat sources. i,j) In the external heat source experiment, schematic diagram of thermal radiation wave propagation inside fabrics cubes. k) The relative shielding efficiency of the metafabric under three heat source frequencies.

However, in radiant heat transfer experiments, metafabric shows a completely different transfer mode. To eliminate the interference of conduction and convection, we place each fabric between the heating device and the aluminum (Al) sheet (**Figure** **4**a), and the fabric has no contact with the heating device and the Al sheet (to avoid conduction), while the ambient temperature remains stable to prevent convection. By detecting the temperature change of Al sheet, the radiation heat transfer mode of each fabric was explored. The results showed that, compared with heating Al sheet directly through air, the Al sheet above the metafabric heats fastest, even in the early stage of heating (Figure 4b and Figure S11, Supporting Information). It is as if the heat bypassed the metafabric and heated the Al sheet directly in the form of thermal radiation (Figure 4c,d). We attribute this phenomenon to the metamaterial properties exhibited by the regular array design of molecular resonant ring of the PPy on the fabric surface (due to linear alignment, planarization and the addition of resonance bridges (MWCNTs). Thus, when heat act on the metafabric in the form of radiated EM wave, the left‐handed property of the metafabric causes the thermal energy propagates on the fabric surface, so it has a high emissivity of 0.992 (close to 1) and low transmittance of 1 × 10^−3^ in the range of 0.2–25 µm (Figure 4e). To exclude the influence of other factors on the experiment results, measurements were carried out on the variation of size and resistance of samples with temperature (Figures S12 and S13, Supporting Information). At the same time, the thermal stability of metamaterials was also tested^[^ ^68^, ^69^ ^]^ (Figure S14, Supporting Information). The results indicated that heat shrinkage phenomenon of metafabric ensures the resistance remained stable in the temperature from 300 to 500 K, which can guarantee the permittivity property stability in EM.

In order to further prove the performance of high‐frequency EM stealth function of the metafabric, different fabrics were woven into a closed square cube, the exterior of the cube was heated with different heat sources (represent three different frequencies range of radiating EM waves) and the temperature changes inside the cube was measured at different times. The temperature rise curve inside the cube reveals that the inner part of the metafabric has the slowest temperature rise and shows better heat shielding properties (Figure 4f,h and Video S1, Supporting Information), which is related to the unique array of regulated the ultra‐small‐sized molecular resonant rings. However, this shielding effect does not allow for an accurate evaluation of the stealthiness of the metafabric against radiated EM waves, as it is very difficult to accurately calculate the energy of EM wave on fabrics. To solve this problem, we take the black cotton fabric as the benchmark and obtain the relative energy absorbed by each fabric through the relationship between the temperature difference and the corresponding energy, and then calculate the thermal shielding efficiency by the EM wave creation. Calculations show that the metafabric shields at least half of the EM energy under the same conditions compared to black cotton fabric. According to the theory of EM wave stealth of metamaterial, the EM wave is propagated along the surface of the metafabric under the action of the molecular resonance rings (Figure 4i,j). As a result, calculation of the thermal shielding efficiency relative to the black cotton fabric also reveal that the metafabric is stealthy to three different frequencies of heat sources. (Relative shielding efficiency: solar energy: 67.12%, mid‐infrared: 51.27%, far infrared: 56.91%; Figure 4k).

**Figure 5:**
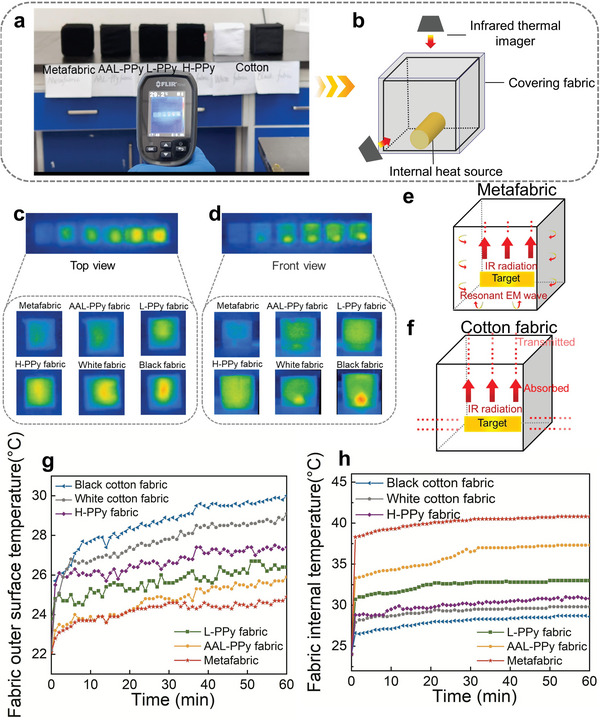
Diagram of the thermal shielding phenomenon of metafabric. a) The physical picture of the stealth performance of the heat source placed inside fabrics cubes with an infrared imager. b) Schematic diagram for determining the stealth performance of heat source placed inside the fabric cubes with an infrared imager. c,d) Top view and front view of infrared image of heat source inside fabric cubes measured by infrared imager. e,f) Heat transfer diagram for metafabric and cotton fabric cubes in the internal heat source experiment. g,h) Curve of the external and internal temperature of the fabric cubes as a function of time when heat source is placed inside the fabric cubes.

As the PPy molecular resonant ring array structure is enriched on both side of the fabric, the EM wave propagation trajectory from either side of the fabric should reflect the properties of the metamaterial. Therefore, in order to observe the effect of metafabric on the radiant EM wave more visually, we changed the position of heat source and placed it inside the cubes with different fabrics. Then use infrared imager and thermocouple to observe the temperature change of the outer and the inner surfaces of the cubes (**Figure** **5**a,b). Surprisingly, the metafabric is almost integrated into the environment, and the infrared imager can hardly detect the heat source inside metafabric cube, whether it is viewed from the front or the top (Figure 5c,d and Video S2, Supporting Information). It feels like there is no heater in the middle of metafabric cube, but in fact, the temperature of the inner surface of metafabric cube is higher than the any other cubes. This further indicates that the EM wave generated by thermal radiation can only be circularly transmitted on the inner surface of the metafabric (Figure 5e,f, Supporting Information). When it acts on the inner surface of the metafabric, resulting in high temperature on the inner environment and low temperature on the outer surface (Figure 5g,h). This indicates that our proposed molecularly sized resonant ring‐structured metamaterial has the ability to stealth EM waves in a wider frequency band through the rational arrangement of the molecular resonant ring unit structure.

## 3 Conclusions

In this study, we demonstrated EM metamaterial designed as connected array of molecular‐level SRR has stealth capabilities at wider frequency EM waves in 10^6^ ∼10^16^ Hz. Theoretically, the metafabric with the array of PPy molecular resonant ring of 2.49 Å diameter has the ability to stealth EM weak up to UV frequencies and even part of the X‐ray frequency range. Although it is not enough to measure the  EM stealth performance of metafabric at UV and X‐ray frequency with the limited characterization method, we are confident to find a suitable method to verify it in future work.

Nowadays, although metamaterials have perfected absorption and shielding capabilities in aircraft stealth, satellite flat panel antennas, and mini base station radio frequency filters, there is still a need for continuous improvement in self‐response adjustment to accommodate variable frequencies, smaller resonant ring designs suitable for high‐frequency stealth and more accurate processing techniques. This research opens up new ideas for the development of metamaterials. This adaptive, molecular‐size resonant ring, which can be arbitrarily shaped, can be used in a number of applications such as solar radiation protection in space (anti‐radiation spacesuits, aircraft coatings), multi‐band radar stealth coatings for aircraft, multi‐band counter‐information protection, thermal radiation stealth for man‐portable combat, UV protection for building, anti‐X‐ray coating protection, etc. This idea of molecular resonant rings and the exciting experimental results pave the way for metamaterials design of structure unit at the molecular level. Of course, there is still a lot of work to be done on metamaterials to achieve applications in these fields, such as 1) The adaptability and reliability of metamaterials under service conditions to be studied; 2) How to obtain metamaterial properties without sacrificing other properties is the key; 3) Metamaterials in low‐cost, multifunctional, and intelligent aspects to be developed.

## 4 Experimental Section

### Materials

The raw materials for the preparation of the sample include Pyrrole (Py, Sigma–Aldrich), FeCl_3_ (Aladdin), Sodium dodecyl benzene sulfonate (SDBS, Aladdin), Multi‐walled carbon nanotubes (MWCNTs, Aladdin), Sodium hydroxide (NaOH, Rhawn), Polyacrylic acid (PAA, Aladdin), Absolute ethanol (Aladdin), Cotton fabric (160 g m^2^, weaving).

### Fabrication of Metafabric

Before pyrrole polymerization on the cotton fabric, the fabric was soaked in NaOH (10 g L^−1^) solution for 30 min and then washed repeatedly with distilled water to remove the residual NaOH solution on the surface. Next, different cotton fabrics were soaked in a reaction vessel containing Py (60 mL, 0.5 mol L^−1^) dispersion for 30 min. Finally, FeCl_3_ (10 mL, 1.5 mol L^−1^) solution was slowly added to the reaction vessel of the fabric and polymerized for 1–5 h at various temperatures (−15 to 60 °C). Specifically, the samples were named according to the polymerization condition shown in **Table** **2**. The sample fabric was washed repeatedly with absolute ethanol and distilled water and dried in an air blast oven at 50 °C.

**Table 2:** Process of different sample preparation

Sample name	H‐PPy	L‐PPy	AAL‐PPy	Metafabric
**Pyrrole Polymerization** **Condition**	Cotton fabric treated by “a” method and polymerization at 60 °C.	Cotton fabric treated by “a” method and polymerization at 0 °C.	Cotton fabric treated by “a‐b” method and polymerization at 0 °C.	Cotton fabric treated by “a‐b‐c” method and polymerization at 0 °C.

Cotton fabric was treated by different combinations of a) NaOH (10 g L^−1^); b) PAA (1 mol L^−1^) solution; c) MWCNTs (2.5 mg L^−1^) and SDBS (10 mg L^−1^) mixed dispersion.

All polymerization using FeCl_3_ (1.5 mol L^−1^) and Py (0.5 mol L^−1^).

To make a comparation, PPy powders were polymerized at the same condition of H‐PPy.

### Characterization

Scanning electron microscope (SEM) images were taken on a Phenom XL. FTIR spectra were recorded by an FTIR spectrophotometer (Nicolet iS50). XPS spectra were measured on an X‐ray photoelectron spectrometer (K‐alpha). The spot size for XPS was 400 µm, the step size was 0.100 eV, and peak fitting method was performed using PeakFit software. XRD patterns were measured on a diffractometer (Rigaku Ultima IV) with Cu‐Ka radiation operated at 40 kV and 30 mA. The sample was scanned in the 2*θ* range of 5–90°. Dielectric properties were investigated by an LCR meter (Agilent, E4991B) equipped with a dielectric detector (Agilent, 16453A) at the frequency of 1 MHz to 1 GHz at room temperature. Permeability properties were investigated by an LCR meter (Agilent, E4991A) equipped with a permeability detector (Agilent, 16454A) at the frequency of 1 MHz to 1 GHz at room temperature. The thermal expansion coefficient of the fabric was measured by TA TMA Q400. The temperature coefficient of fabric resistance is measured by RT module of PPMS (Quantum Design). Thermogravimetric measurement (TGA Netzsch TG 209 F3) was conducted at a heating rate of 10 °C min^−1^ under airflow to demonstrate the thermal stability.

### Thermal Measurements of the PPy Fabrics and Metafabric

The metafabric is pasted on a pair of wooden strips with certain spacing with polyimide tape, and the Al foil cut with the same size as the metafabric is pasted on the wooden strips with polyimide tape. Fix the two groups of wooden strips up and down. Attach polyimide tape to the thermocouple on the surface of Al foil, and the thermocouple thermometer (JK804) records its surface temperature. Place the assembled wooden strip on the heating table and set the temperature to 100 °C. Different fabrics are sewn with cotton thread into 10 × 10 cm^2^ size closed cube frame. A thermocouple thermometer is suspended in the middle to test the ambient temperature in the frame. Place the cube frame under a medium infrared lamp (0.76–5 µm), a solar simulator (AM1.5), and a heating table (100 °C). Emissivity and transmissivity spectra were measured in the 0.2–2.5 µm range using a spectrophotometer (SPC UV‐3600/UV‐3600 iplus) with an integrating sphere. A Fourier transform infrared spectrometer (Nicolet iS50) equipped with an integrating sphere was measure the IR emissivity and transmissivity in the 2.5–25 µm range.

### Comsol Multiphysics Numerical Simulation

Real materials were used to discuss the 3D situation, and EM waves and frequency‐domain physical fields were applied. And the left port input transverse current (TE), modulus was 10. The top and bottom boundaries were electrically insulated. It could be seen that ordinary fabrics have no shielding effect in terms of electricity and magnetism, and EM waves could directly pass through the cube fabric in the middle. The cube frame woven by metafabric can guide EM waves around the cube itself, and EM waves cannot enter the interior of the cube. With the progress of technology, the shielding effect was getting better and better, and the metafabric has the best EM shielding effect (Figures S6–S10, Supporting Information).

### Calculation of Relative Heat Radiation Shielding Efficiency

It could be seen from this group of data that the relative shielding efficiency of metafabric was the highest under the irradiation of three different wavelength heat sources (Tables S5 and S6, Supporting Information). It shows that metafabric has the most excellent ability to shield radiant heat. In addition, the relative radiation shielding efficiency of the five samples irradiated by the medium infrared lamp heat source was basically the same as that of the heating table heat source (far infrared wavelength), and was lower than that of the samples irradiated by the sunlight heat source. This is because the wavelength range covered by sunlight was wider, which was more suitable for the work of metafabric.

Based on Fourier's law, Newton's law of cooling, and the Stefan–Boltzmann law, the heat flux terms could be expressed as shown in Equation (1), (2), (3),

Sunlight simulator:

(1) $$\begin{array}{ccc} Q\; & = & \;3\%\cdot \left(1-\tau_{uv}-\rho_{uv}\right)P_{sun}+45\%\cdot \left(1-\tau_{vis}-\rho_{vis}\right)P_{sun}\\ & + & 52\cdot \left(1-\tau_{nir}-\rho_{nir}\right)P_{sun} \end{array}$$

Mid infrared emitting bulb:

(2) $$Q\;=\;\left(1-\tau_{mir}-\rho_{mir}\right)\frac{d{\alpha P}_{bulb}}{\mathrm{dS}}$$

Heating table heat source:

Radiant heat radiated from the surface temperature of the heating table to the fabric:

(3) $$Q\;=\;\left(1-\tau_{fir}-\rho_{fir}\right)\left(\frac{\left(T_{h}^{4}-T_{to}^{4}\right)}{\frac{1}{\varepsilon_{h}}+\frac{1}{\varepsilon_{mir}}-1}\right)$$

where *Q* is heat source energy incident on the internal environment of fabric, *ρ*_*uv*_ and *τ*_*uv*_ are the weighted average reflectance and transmittance of the fabric to ultraviolet wavelengths, respectively. *ρ*_*vis*_ and *τ*_*vis*_ are the weighted average reflectance and transmittance of the fabric to visible wavelengths, respectively. *ρ*_*nir*_ and *τ*_*nir*_ are the weighted average reflectance and transmittance of the fabric to near infrared wavelengths, respectively. *ρ*_*mir*_ and *τ*_*mir*_ are the weighted average reflectance and transmittance of the fabric to mid infrared wavelengths, respectively. *ρ*_*fir*_ and *τ*_*fir*_ are the weighted average reflectance and transmittance of the fabric to far infrared wavelengths, respectively. *ε*_*h*_ is emissivity of heating table. *α* is photoelectric conversion efficiency of bulb. *P_sun_* is solar irradiance (1000 W·m^−2^), *P_bulb_* is rated power of bulb (100 W). S is surface area of fabric receiving bulb. *T_h_* is surface temperature of heating table, *T_to_* is outer surface temperature of fabric. See Table S7 (Supporting Information) for detailed parameters.

Due to the limited experimental conditions, only the shielding efficiency of fabrics with metafabrics and other polymerization conditions compared with cotton fabrics could be calculated, which is called the relative shielding efficiency of radiant heat (*η*), as shown in Equations (4), (5), (6).

(4) $$\eta =\frac{Q_{s}}{Q}$$

(5) $$Q_{S}=Q-Q^{\prime }$$

(6) $$Q^{\prime }=\Delta T\cdot \Delta P$$

where *Q* is energy obtained by fabric (W m^−2^), Δ*T* is difference between internal temperature of fabric cube and ambient temperature (K), *Q*′ is theoretical energy required to cause the change of Δ*T* (W m^−2^), Δ*P* is the energy required by cotton fabric for every 1 K rise, which is taken as the standard for other conditional fabrics (K (W m^−2^) ^‐1^), *Q_s_* is energy of fabric shielding (W m^−2^).

## Conflict of Interest

The authors declare no conflict of interest.

## Supporting information

Supporting Information

Supplemental Video 1

Supplemental Video 2

## Data Availability

Research data are not shared.
