# Supplementary material for: Molecularly Resonant Metamaterials for Broad‐Band Electromagnetic Stealth
Source: Adv Sci (Weinh). 2023 Apr 21;10(19):2301170. doi: 10.1002/advs.202301170 (PMC10323625; doi:10.1002/advs.202301170)
Supplement: Supplementary file 1 — Supporting Information [file ADVS-10-2301170-s003.pdf]

ADVANCED SCIENCE
Open Access

Supporting Information

for *Adv. Sci.*, DOI 10.1002/advs.202301170

Molecularly Resonant Metamaterials for Broad-Band Electromagnetic Stealth

Yifan Wang, Jiarong Niu, Xin Jin, Xiaoming Qian, Changfa Xiao and Wenyu Wang**

Supplementary Information for

Molecularly resonant metamaterials for broad-band electromagnetic stealth

Yifan Wang†, Jiarong Niu†, Xin Jin*, Xiaoming Qian, Changfa Xiao, Wenyu Wang*

This PDF file includes:

Supplementary Discussion

Figure S1-S14

Table S1-S6

Other Supplementary Materials for this manuscript include the following:

Videos S1-S2 (.mov)

Supplementary Discussion

Proof of heat conduction and convection experiments.

In order to prove that the increase of the internal temperature of the five fabrics is mainly caused by the difference of radiant heat, two groups of experiments were conducted to eliminate the interference of heat convection and heat conduction on the fabrics. The schematic diagram is as shown in the Figure S10. The data is temperature rise of cotton fabric/temperature rise of other fabrics (Table S4). The first experiment is to use heat conduction as the main heat transfer method to heat the fabric. This column of data fluctuates between 0.89-1, indicating that the difference in heat conduction has little impact on the five fabrics. In the second experiment, the fabric was heated mainly by heat convection. The data fluctuated between 1.0359-1, indicating that the difference of heat convection has little effect on the five fabrics. To sum up, in the case that both heat conduction and heat convection have little influence on the sample fabric, only radiant heat has great influence on the fabric. Therefore, this also proves that the difference in the internal temperature of the five fabrics is mainly caused by the difference in radiant heat.

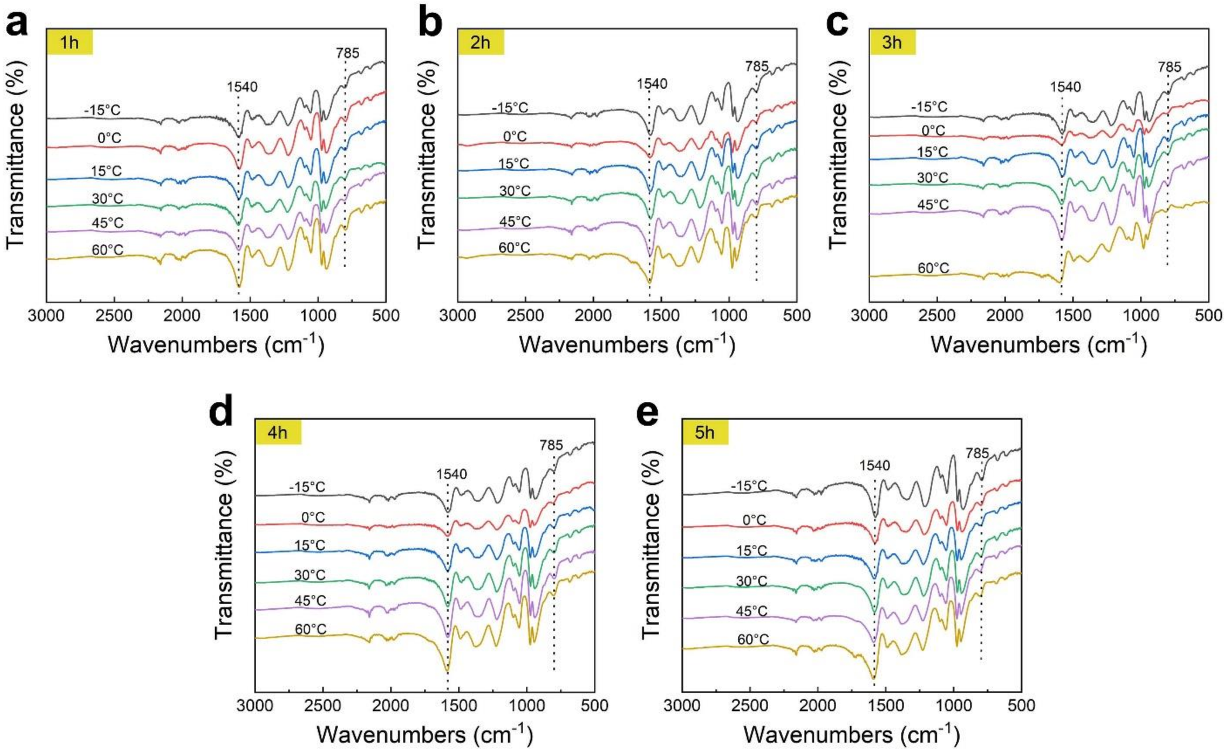

Figure S1. FTIR spectra of polymeric powders under different conditions. FTIR spectra of polymeric powders under different conditions. Infrared spectra of powders polymerized at different temperatures for (a) 1h, (b) 2h, (c) 3h, (d) 4h and (e) 5h respectively.

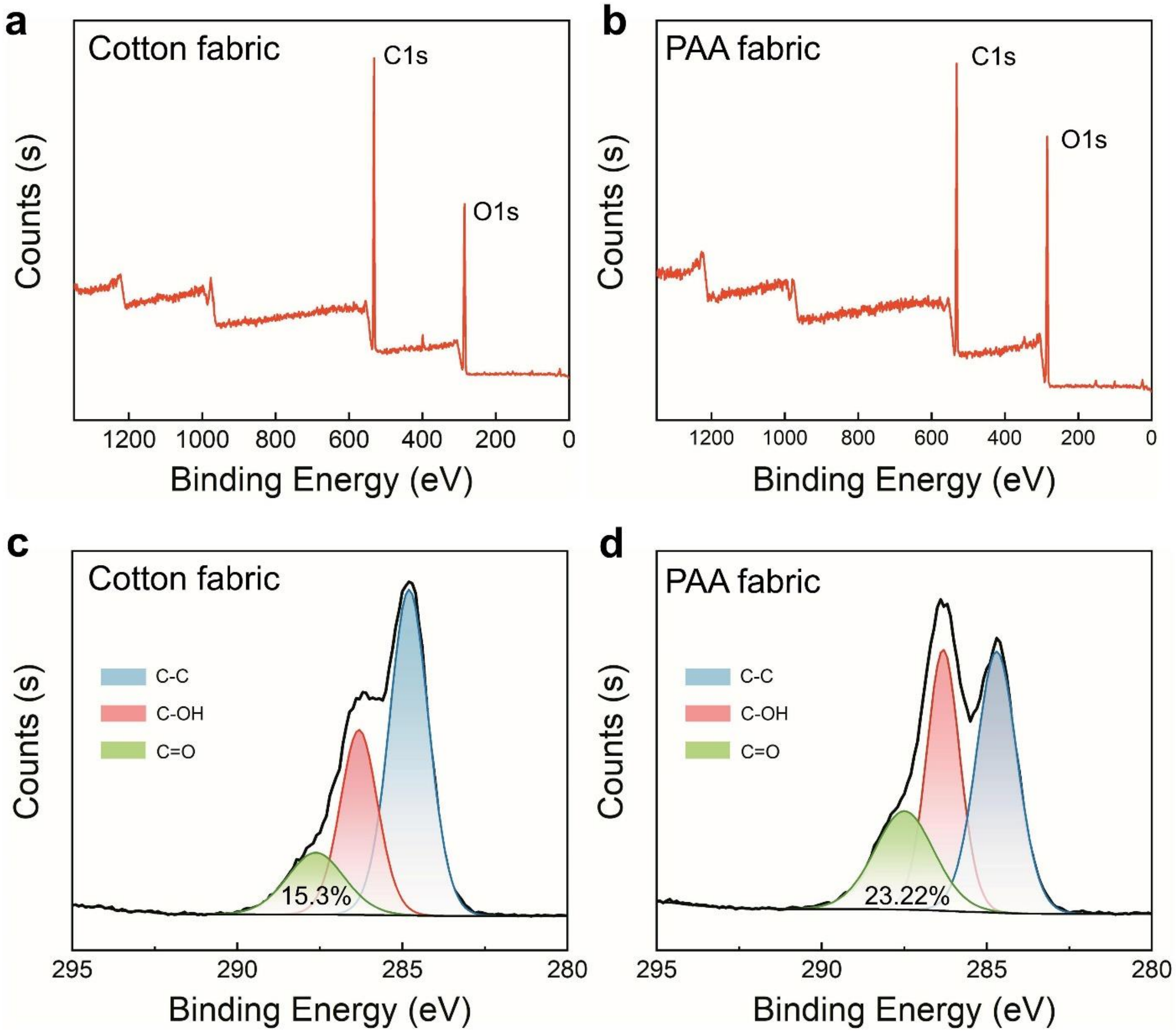

Figure S2. C1s XPS spectra and the fitted C1s XPS spectra of polymeric fabrics under different conditions. C1s XPS spectrum of (a) Cotton fabric and (b) PAA-fabric respectively. The O content of cotton fabric after polyacrylic acid treatment is significantly increased. Fitted C1s XPS spectrum of (c) Cotton fabric and (d) PAA-fabric respectively. The -COOH group content of cotton fabric and PAA fabric are 15.3% and 23.22%. It can be proved that the cotton fabric substrate has been successfully grafted with -COOH group.

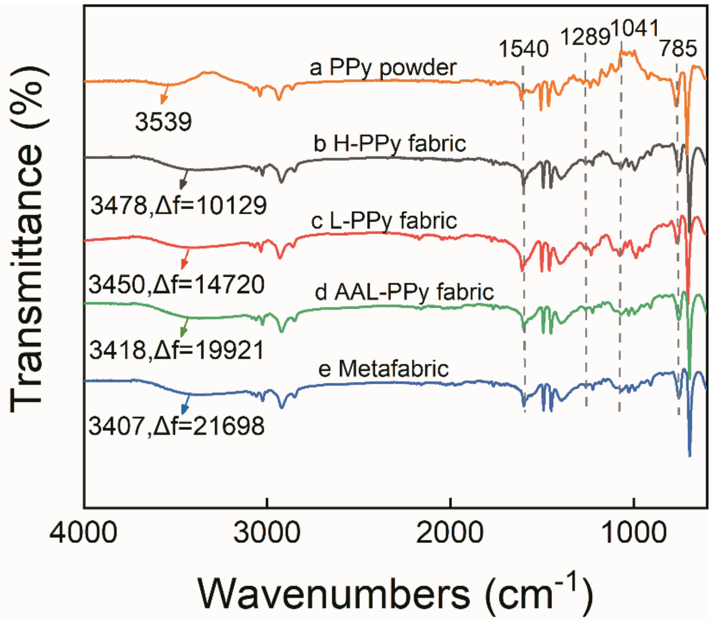

Figure S3. FTIR spectra of polymeric fabrics under different conditions. Infrared spectra of (a) PPy powder, (b) H-PPy fabric, (c) L-PPy fabric, (d) AAL-PPy fabric and (e) Metafabric respectively. To make a study on the molecular interaction in PPy composite electrode, the reduction in force constant *Δf* from FTIR is adopted to evaluate the interaction between the sample and PPy powders, which was calculated based on -NH shift in FTIR data.

According to the harmonic oscillator model, Δf (reduction in force constant) was calculated by following equation based FTIR data as shown in Equation S1.

$$\Delta \mathrm{f} = \frac{\mu(v_2^2 - v_1^2)}{4\pi^2} \qquad \text{(S1)}$$

Where $\mu = \frac{m_1 m_2}{m_1 + m_2}$ corresponding to the reduced mass of harmonic oscillator, v_1 and v_2 are the oscillating frequency of bonded and non-bonded oscillator.

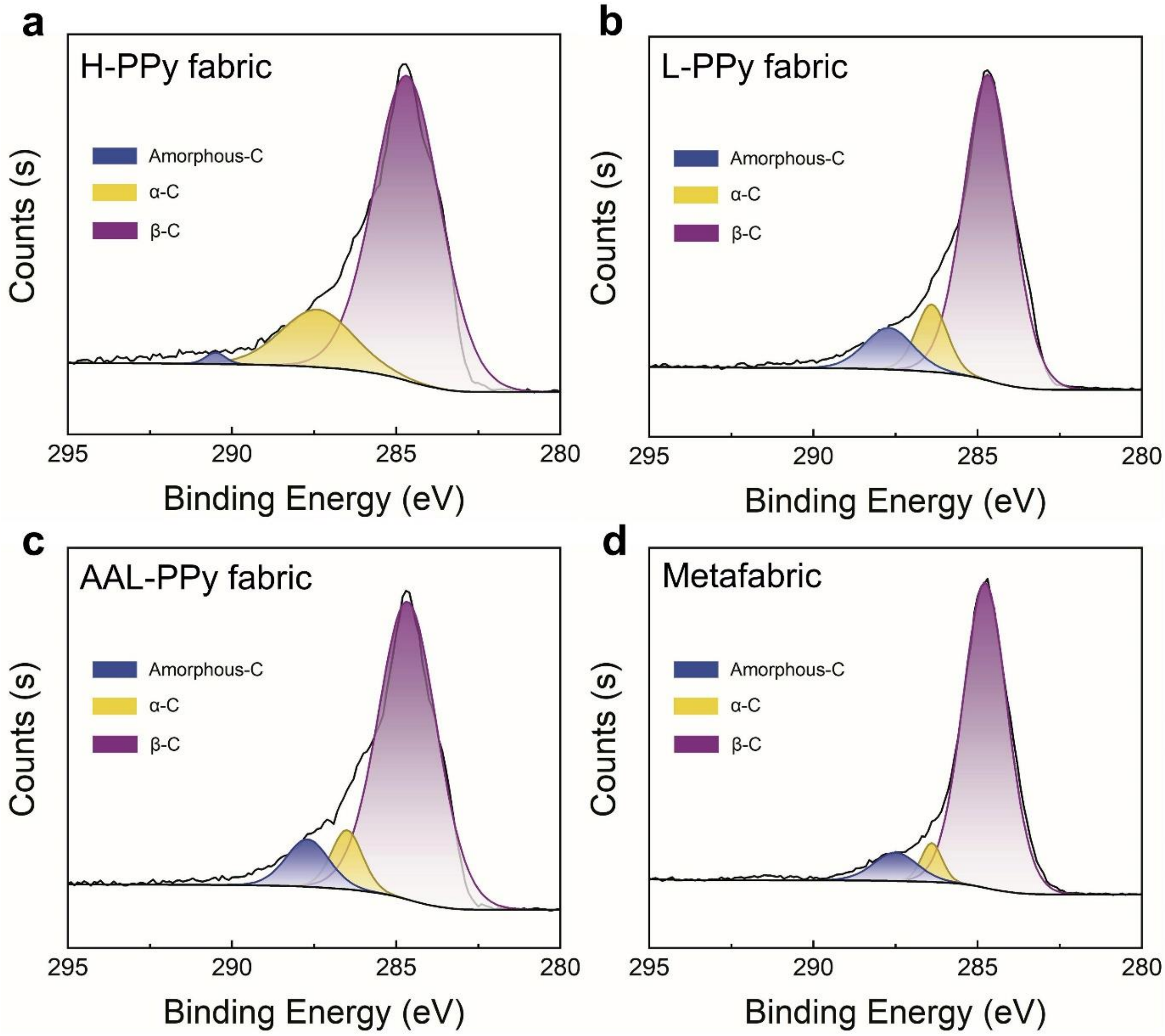

Figure S4. The fitted C1s XPS spectra of polymeric fabrics under different conditions. The fitted C1s XPS spectra of (a) H-PPy fabric, (b) L-PPy fabric, (c) AAL-PPy fabric and (d) Metafabric. The fitted C1s peaks associated with binding energies at 285.30 eV, 286.27 eV, and 288.04 eV corresponded to β-C, α-C and amorphous C, respectively.

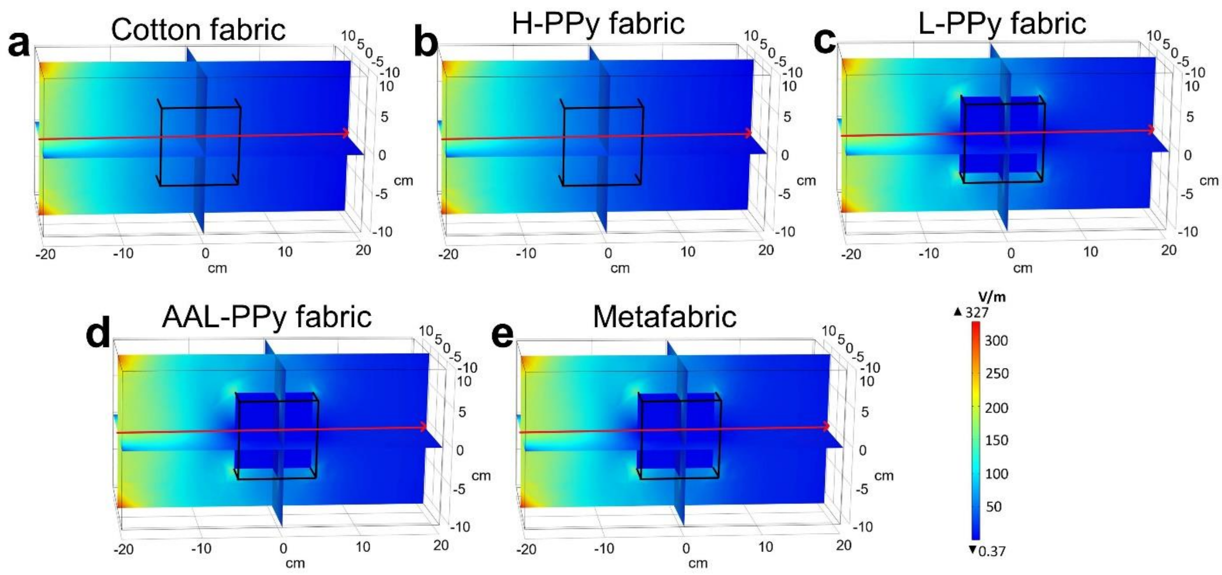

Figure S5. E-field distribution in xyz three-dimensional free space. Shielding electric field distribution of (a) Cotton fabric, (b) H-PPy fabric, (c) L-PPy fabric, (d) AAL-PPy fabric and (e) Metafabric cube at 1GHz.

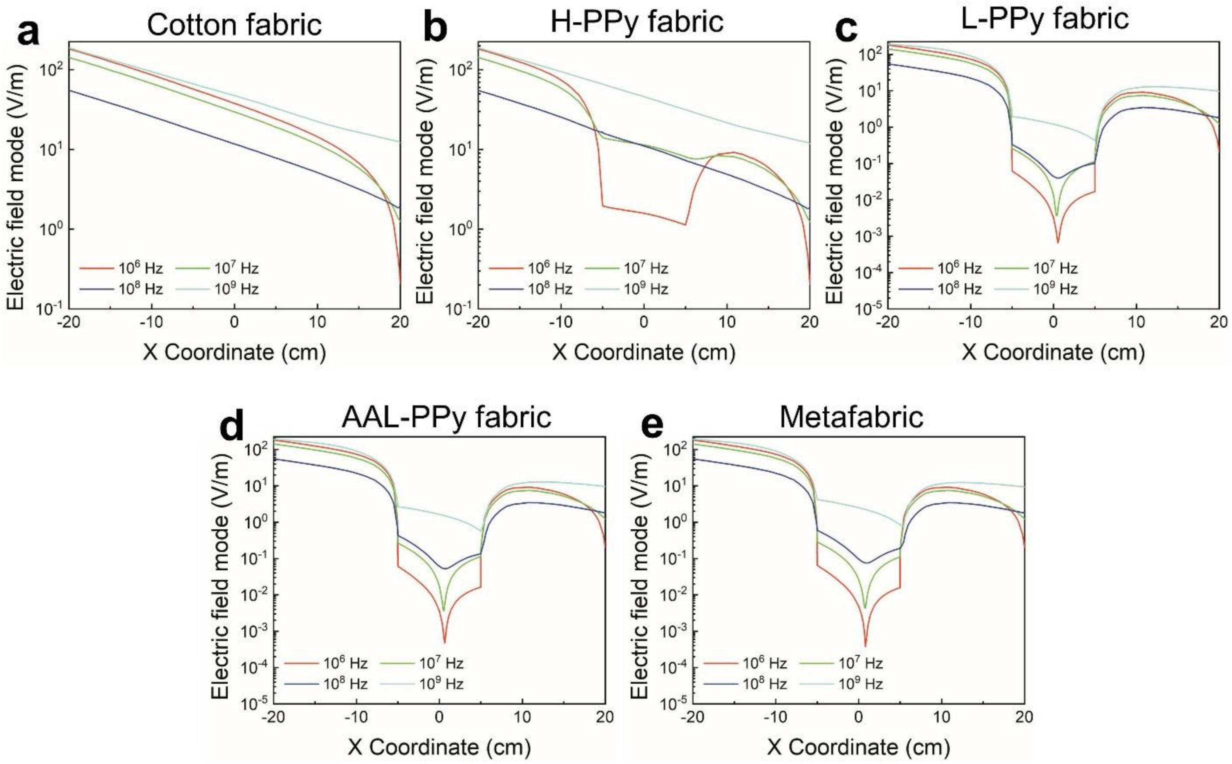

Figure S6. E-field line distribution of x-axis transverse line. Distribution of electric field lines along the x-axis transverse section of (a) Cotton fabric, (b) H-PPy fabric, (c) L-PPy fabric, (d) AAL-PPy fabric and (e) Metafabric cube.

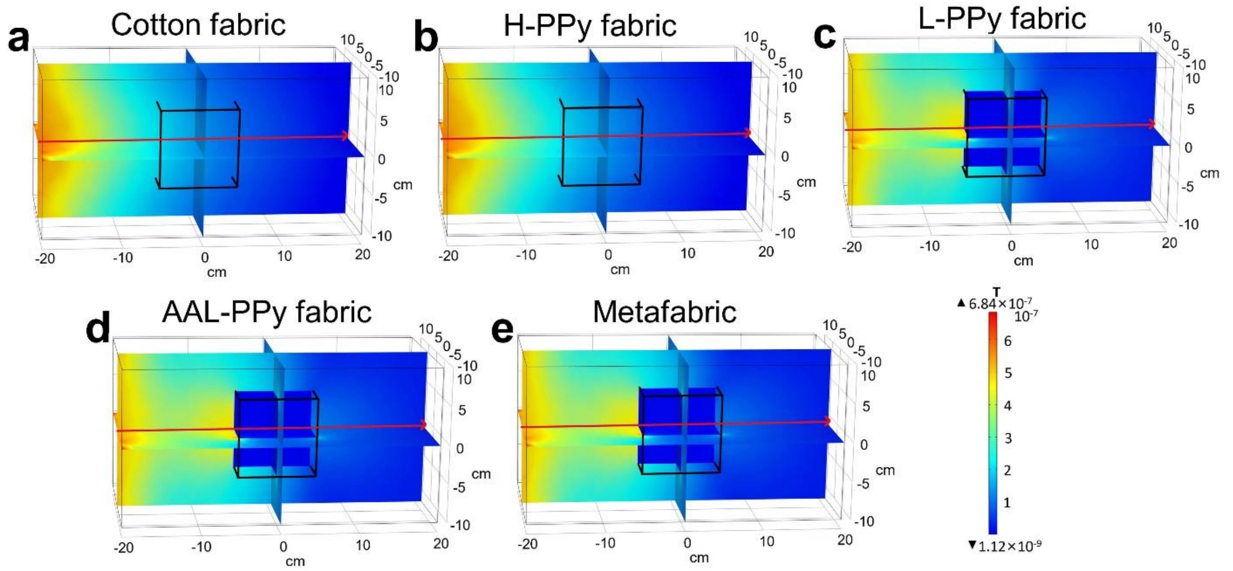

Figure S7. H-field distribution in xyz three-dimensional free space. Shielding magnetic field distribution of (a) Cotton fabric, (b) H-PPy fabric, (c) L-PPy fabric, (d) AAL-PPy fabric and (e) Metafabric cube at 1GHz.

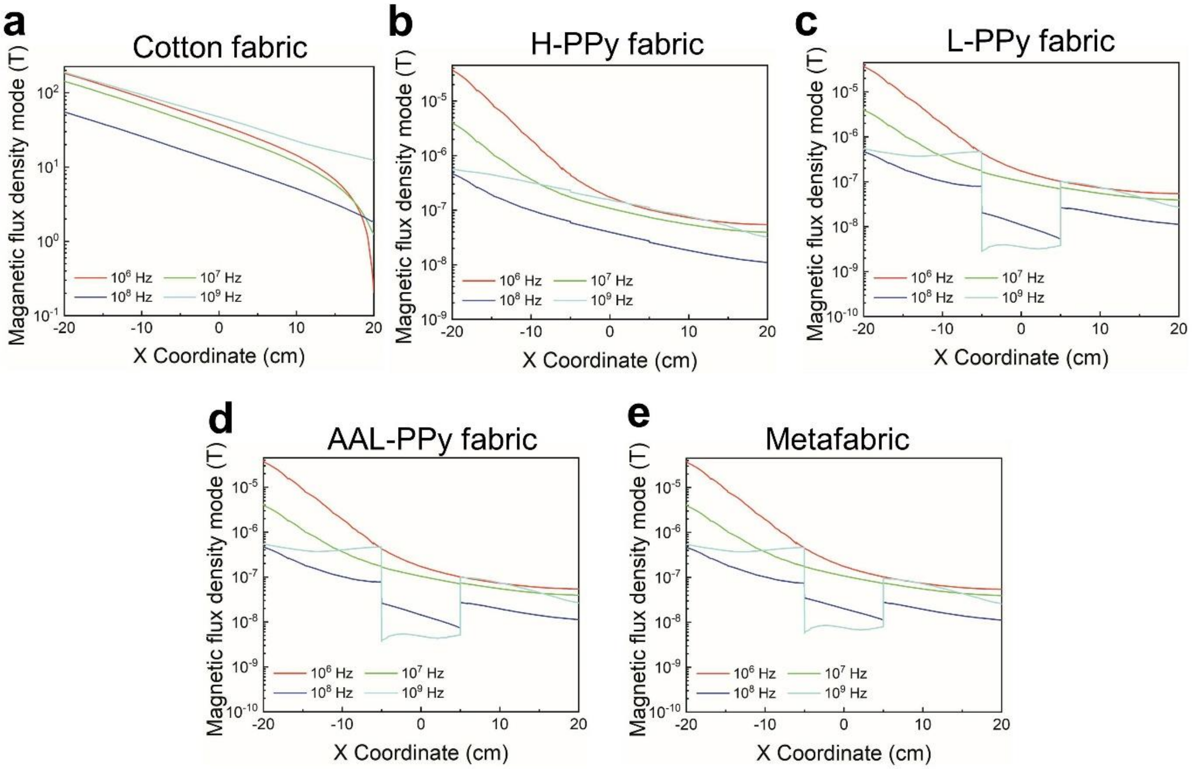

Figure S8. H-field line distribution of x-axis transverse line. Shielding magnetic field distribution of (a) Cotton fabric, (b) H-PPy fabric, (c) L-PPy fabric, (d) AAL-PPy fabric and (e) Metafabric cube at 1MHz-1GHz.

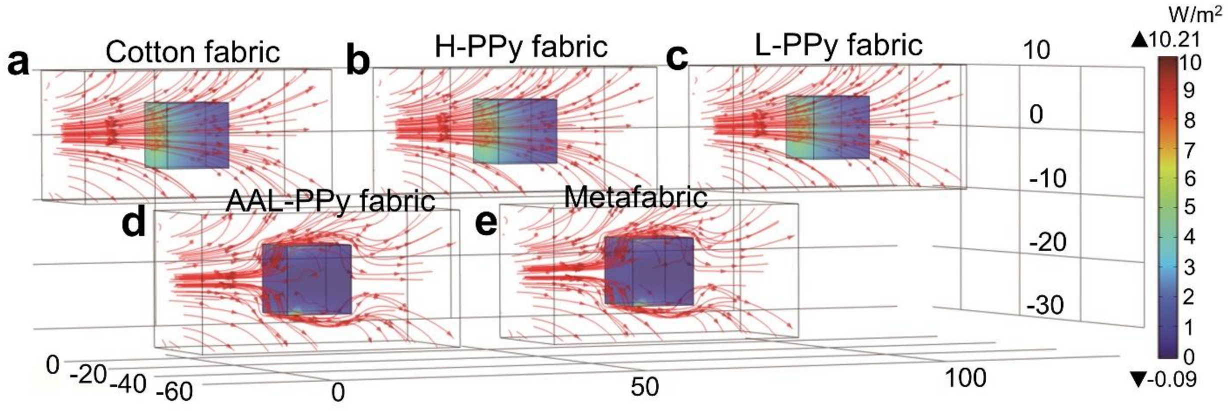

Figure S9. Time average power flow distribution diagram. Time averaged power flow distribution of (a) Cotton fabric, (b) H-PPy fabric, (c) L-PPy fabric, (d) AAL-PPy fabric and (e) Metafabric cube at 1GHz.

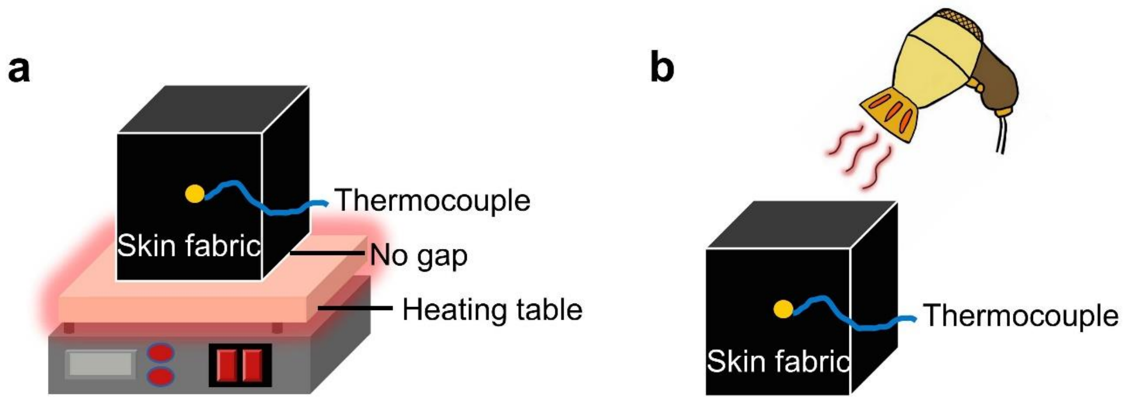

Figure S10. Schematic diagram of the experiment. (a) Schematic diagram of heat conduction experiment, (b) Schematic diagram of thermal convection experiment.

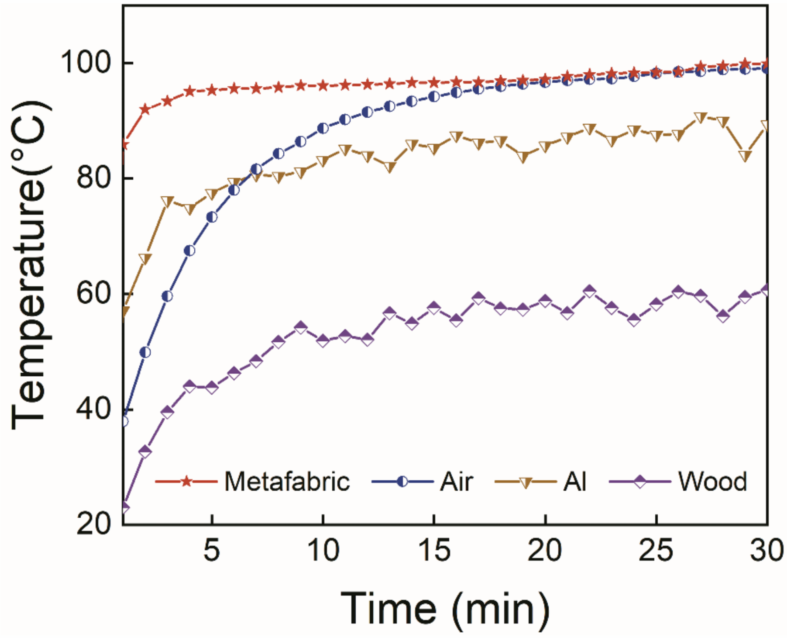

Figure S11. The time temperature curve of the medium is metafabric and other materials.

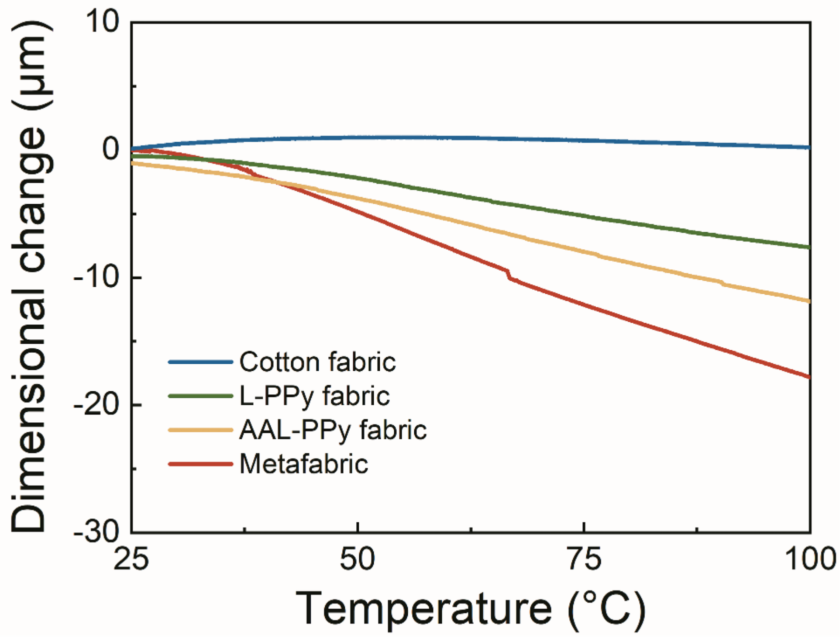

Figure S12. With the increase of temperature, the expansion rate of different PPy fabrics changes.

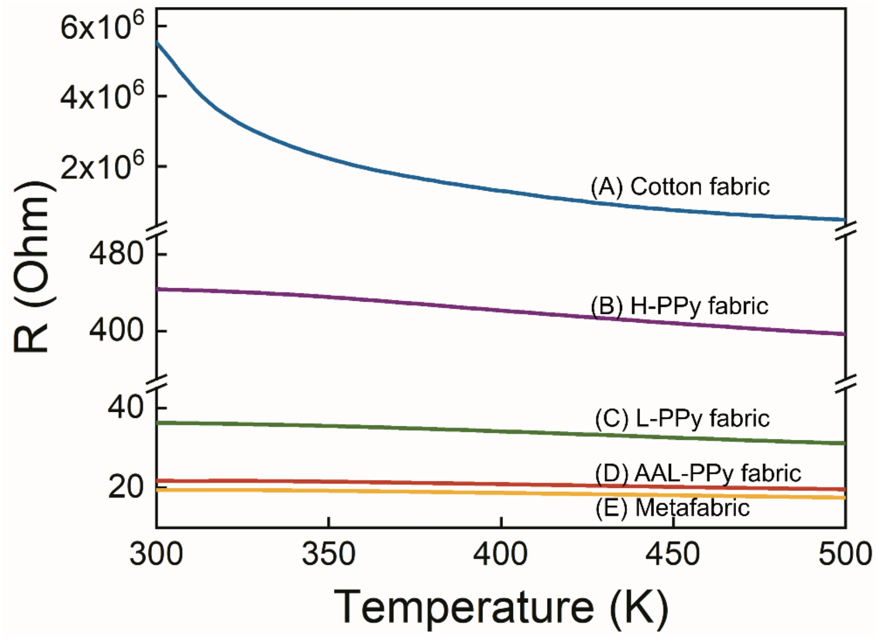

Figure S13. Temperature coefficient variation of electrical resistance of different fabrics.

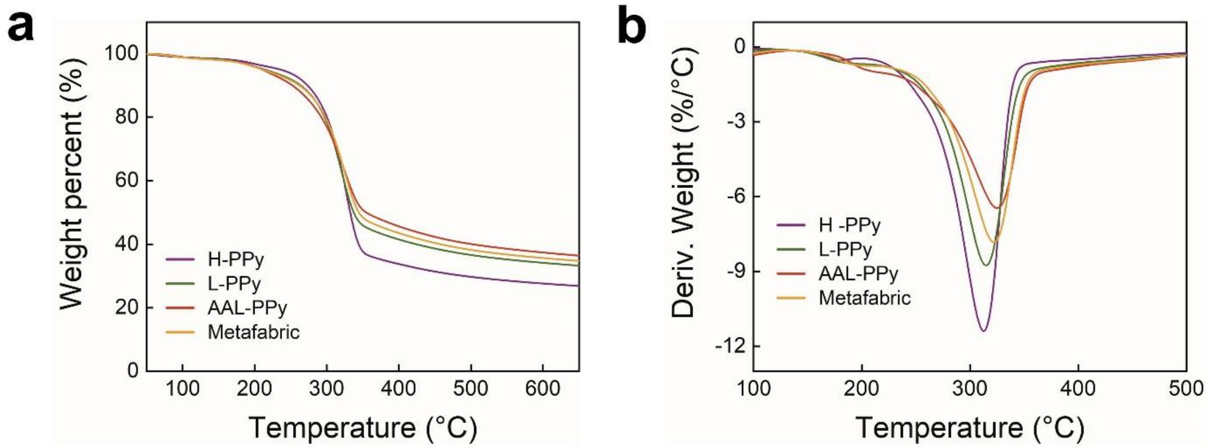

Figure S14. (a) TGA curves of different fabrics, (b) DTG curves of different fabrics. The peak temperature of the DTG represents the maximum weight loss temperature. This result indicates that Metafabric has better thermal stability.

Table S1. Infrared I_{785} /I_{1540} ratio under different polymerization conditions.

Temperature/ ℃ Time/h	-15	0	15	30	45	60
**1**	0.6553	0.6705	0.6622	0.6759	0.6536	0.6031
**2**	0.6832	0.7391	0.6860	0.6954	0.6279	0.5226
**3**	0.7071	0.7743	0.7418	0.6343	0.5992	0.4825
**4**	0.7135	0.7704	0.6961	0.6275	0.5902	0.4778
**5**	0.7418	0.7367	0.6309	0.6174	0.5081	0.4237

Table S2. Infrared I_{785} /I_{1540} ratio under different polymeric fabrics.

**Sample**	**H-PPy fabric**	**L-PPy fabric**	**AAL-PPy fabric**	**Metafabric**
**α-α**	0.4465	0.7664	0.9579	0.9601

Table S3. The $\alpha_{area}/\beta_{area}$ content under different polymeric fabrics.

Sample	H-PPy fabric	L-PPy fabric	AAL-PPy fabric	Metafabric
$\boldsymbol{\alpha_{area}/\beta_{area}}$	0.2051	0.1409	0.1058	0.0604

Table S4. Data from heat conduction and convection experiments.

Heat transfer mode / Sample	Heat conduction	Thermal convection
**Metafabric**	0.8951	1.0359
**AAL-PPy fabric**	0.9070	1.0375
**L-PPy fabric**	0.9223	1.0440
**H-PPy fabric**	0.9349	1.0540
**Cotton fabric**	1	1

Table S5. Calculation of relative shielding efficiency of radiant heat.

**Sunlight**	**Meta fabric**	**AAL-PPy fabric**	**L-PPy fabric**	**H-PPy fabric**	**Cotton fabric**	$\boldsymbol{P}$ **$[K\cdot(W\cdot m^{-2})^{-1}]$**
$\boldsymbol{Q}$ **$[(W\cdot m^2)^{-1}]$**	690.1	661.4	638.6	605.8	531.4	59.71
$\boldsymbol{\Delta T}$ **[℃]**	3.8	4.9	6.2	7.3	8.9	
$\boldsymbol{Q'}$ **$[(W\cdot m^2)^{-1}]$**	226.898	292.579	370.202	435.883	531.4	
$\boldsymbol{Q_s}$ **$[(W\cdot m^2)^{-1}]$**	463.202	368.821	268.398	169.917	0	
$\boldsymbol{\eta}$ **[%]**	67.12	55.76	42.03	28.05	Measurement standard	
**Mid infrared**	**Meta fabric**	**AAL-PPy fabric**	**L-PPy fabric**	**H-PPy fabric**	**Cotton fabric**	15.68
$\boldsymbol{Q}$ **$[(W\cdot m^2)^{-1}]$**	435.4	425.2	415.4	403.6	373.2	
$\boldsymbol{\Delta T}$ **[℃]**	13.6	14.8	15.9	18.5	23.8	
$\boldsymbol{Q'}$ **$[(W\cdot m^2)^{-1}]$**	212.16	232.064	249.312	290.08	373.184	
$\boldsymbol{Q_s}$ **$[(W\cdot m^2)^{-1}]$**	223.24	193.136	166.088	113.52	0	
$\boldsymbol{\eta}$ **[%]**	51.27	45.42	39.98	28.13	Measurement standard	
**Far infrared**	**Meta fabric**	**AAL-PPy fabric**	**L-PPy fabric**	**H-PPy fabric**	**Cotton fabric**	15.61
$\boldsymbol{Q}$ **$[(W\cdot m^2)^{-1}]$**	420.0	414.4	408.7	402.2	380.8	
$\boldsymbol{\Delta T}$	11.6	14.7	18.2	21.2	24.4	

**[°C]**					
***Q'* [(W·m²)⁻¹]**	180.96	229.467	284.102	330.932	380.884
**Q_s [(W·m²)⁻¹]**	239.04	184.933	124.598	71.268	0
***η* [%]**	56.91	44.63	30.49	17.72	Measurement standard

Table S6. Definitions and values of parameters in the relative shielding efficiency of radiant heat.

Symbol	Definition	Value	Unit
σ	Stefan-Boltzmann constant	5.67×10^{-8}	$[W\cdot(m^2\cdot K^4)^{-1}]$
τ_{vis}	VIS transmittance	Metafabric：0.0002<br>AAL-PPy fabric：0.00607<br>L-PPy fabric：0.00619<br>H-PPy fabric：0.01153<br>Black cotton fabric：0.2316	
τ_{uv}	UV transmittance	Metafabric：0.0004<br>AAL-PPy fabric：0.00406<br>L-PPy fabric：0.0060<br>H-PPy fabric：0.01442<br>Black cotton fabric：0.49345	
τ_{nir}	NIR transmittance	Metafabric：0.0002<br>AAL-PPy fabric：0.0038<br>L-PPy fabric：0.0048<br>H-PPy fabric：0.0084<br>Black cotton fabric：0.1930	
τ_{mir}	MIR transmittance	Metafabric：0.000018<br>AAL-PPy fabric：0.000025<br>L-PPy fabric：0.000042<br>H-PPy fabric：0.002227<br>Black cotton fabric：0.0821	
τ_{fir}	FIR transmittance	Metafabric：0.00001<br>AAL-PPy fabric：0.00004<br>L-PPy fabric：0.00006	

		H-PPy fabric：0.00303
		Black cotton fabric：0.08625
$\boldsymbol{\rho_{vis}}$	VIS reflectivity	Metafabric：0.3808
		AAL-PPy fabric：0.38191
		L-PPy fabric：0.38545
		H-PPy fabric：0.42547
		Black cotton fabric：0.25556
$\boldsymbol{\rho_{uv}}$	UV reflectivity	Metafabric：0.51144
		AAL-PPy fabric：0.51194
		L-PPy fabric：0.51781
		H-PPy fabric：0.55658
		Black cotton fabric: 0.10446
		Black cotton fabric：0.10446
$\boldsymbol{\rho_{nir}}$	NIR reflectivity	Metafabric：0.2366
		AAL-PPy fabric：0.2532
		L-PPy fabric：0.2537
		H-PPy fabric：0.3383
		Black cotton fabric：0.252
$\boldsymbol{\rho_{mir}}$	MIR reflectivity	Metafabric：0.008762
		AAL-PPy fabric：0.013455
		L-PPy fabric：0.015858
		H-PPy fabric：0.030853
		Black cotton fabric：0.06832
$\boldsymbol{\rho_{fir}}$	FIR reflectivity	Metafabric：0.00798
		AAL-PPy fabric：0.01122
		L-PPy fabric：0.01455
		H-PPy fabric：0.01696

		Black cotton fabric：0.01435	
$\boldsymbol{\varepsilon_h}$	Heating table emissivity	0.05	
$\boldsymbol{T_{to}}$	Outer surface temperature of fabric	Metafabric：326.6<br>AAL-PPy fabric：327.8<br>L-PPy fabric：328.5<br>H-PPy fabric：329.3<br>Black cotton fabric：331.0	[K]
$\boldsymbol{S}$	Surface area of fabric exposed to heat source	Metafabric：0.01<br>AAL-PPy fabric：0.01<br>L-PPy fabric：0.01<br>H-PPy fabric：0.01<br>Black cotton fabric：0.01	[m^2]
$\boldsymbol{\alpha}$	Photoelectric conversion efficiency of bulb	30	[$lm \cdot W^{-1}$]

Video S1. Under the solar heat source, middle infrared heat source and fir-infrared heat source, the internal temperature of the cotton fabric and metafabric cube changes within 1h. Here, the solar heat source is replaced by a solar simulator (AM 1.5), the middle infrared heat source is replaced by a mid-infrared emission lamp (0.76-5 μm), the fir-infrared heat source is replaced by a heating table (100 ℃).

Video S2. The image detected by the infrared imager when the heat source is placed inside the cube.
